# Simple estimators of the intensity of seasonal occurrence

**DOI:** 10.1186/1471-2288-8-67

**Published:** 2008-10-22

**Authors:** M Alan Brookhart, Kenneth J Rothman

**Affiliations:** 1 Division of Pharmacoepidemiology and Pharmacoeconomics, Department of Medicine, Brigham and Women's Hospital & Harvard Medical School Boston, MA, USA; 2 RTI Health Solutions Research Triangle Park, NC, USA

## Abstract

**Background:**

Edwards's method is a widely used approach for fitting a sine curve to a time-series of monthly frequencies. From this fitted curve, estimates of the seasonal intensity of occurrence (i.e., peak-to-low ratio of the fitted curve) can be generated.

**Methods:**

We discuss various approaches to the estimation of seasonal intensity assuming Edwards's periodic model, including maximum likelihood estimation (MLE), least squares, weighted least squares, and a new closed-form estimator based on a second-order moment statistic and non-transformed data. Through an extensive Monte Carlo simulation study, we compare the finite sample performance characteristics of the estimators discussed in this paper. Finally, all estimators and confidence interval procedures discussed are compared in a re-analysis of data on the seasonality of monocytic leukemia.

**Results:**

We find that Edwards's estimator is substantially biased, particularly for small numbers of events and very large or small amounts of seasonality. For the common setting of rare events and moderate seasonality, the new estimator proposed in this paper yields less finite sample bias and better mean squared error than either the MLE or weighted least squares. For large studies and strong seasonality, MLE or weighted least squares appears to be the optimal analytic method among those considered.

**Conclusion:**

Edwards's estimator of the seasonal relative risk can exhibit substantial finite sample bias. The alternative estimators considered in this paper should be preferred.

## Background

In a classic paper, Edwards [1] describes a geometrically motivated, moment-based method to fit a sine curve to a time series of square-root transformed monthly frequencies. From this basic framework, he derived both a test of the null hypothesis of no seasonality and an estimator of the intensity of seasonal occurrence (i.e., the peak-to-low ratio of the fitted sine curve). Owing to its intuitive appeal and computational simplicity, Edwards's and related methods have been widely used in epidemiology in studies of seasonality, e.g., [2-7].

Although there has been considerable discussion of the hypothesis testing procedure described by Edwards and a variety of alternative tests have been proposed [8-12], there has been relatively little discussion of the properties of Edwards's estimator of the intensity of seasonal occurrence. St. Leger discusses some computational difficulties involved with maximum likelihood estimation of the parameters in Edwards's model [13]. Nam compared the performance of the MLE with a moment-based "locally reasonable" estimator, similar to Edwards's estimator, and concluded that the MLE was preferable when the seasonal trend was strong [14].

In this paper, we review various approaches to the estimation of the intensity of seasonal occurrence, including Edwards's methods, least squares, weighted least squares, and the MLE. We then propose a new closed-form moment estimator of the peak-to-low ratio based on non-transformed data and a second-order moment statistic. Through an extensive Monte-Carlo simulation study, we compare the finite sample performance of the estimators discussed in this paper across a variety of data generating distributions, including some that involve overdispersion and autocorrelation of the outcome and thus depart from the assumed model. All estimators and confidence interval procedures discussed in this paper are applied in a reanalysis of data on the seasonal incidence of monocytic leukemia.

## Methods

### Data and Probability Model

Edwards's approach is used to study the seasonality of rare events that arise from an underlying non-homogeneous Poisson process with a rate given by the periodic function

*λ*(*t*) = *μ*{1 + *α*cos(2*πt *+ *θ*)},

where *μ *is the total number of expected events in the year, *t *is the time in years, *θ *is the phase angle, and *α *is the hemi-amplitude of the periodic process.

We consider the situation in which the year is divided into *k *equally-sized intervals and aggregate data are available on the frequency of events occurring in each interval across *T *years. We denote the observed frequencies with *N*_*i*_, *i *= 1, ..., *k*.

Edwards's probability model for these data is a discrete approximation to the non-homogeneous Poisson process and models the observed counts as independent Poisson random variables with mean given by the periodic function

(1) $$m_{i}=\frac{n}{k}\left\{1+\alpha \cos[\frac{2\pi }{k}(i-\phi -0.5)]\right\},$$

where *i *is the interval (e.g., quarter, month, week), and *φ *+ 0.5 is the time of peak incidence. The parameter n is the total expected number of events across all years, i.e., *n *= *μT*.

In this paper, we focus on the estimation of the peak-to-low ratio of the process, also termed the intensity of seasonal occurrence or seasonal relative risk, and is given by

$$R=\frac{1+\alpha }{1-\alpha }.$$

### Edwards's Method

Edwards derives an estimator for *α *by first computing the distance from the origin to a re-scaled center of gravity of *k *point masses of weight $\sqrt{N_{i}}$ placed on the rim of a unit circle at angles *θ*_*i *_= 2*πi*/*k*, *i *= 1, ..., *k*. Using a first-order Taylor series expansion he derives an expected value for this quantity that depends on the true *α*. Setting the distance from the origin to the center of gravity equal to its expected distance and solving for *α*, Edwards derives a moment-based estimator for a given by

$${\hat{\alpha }}_{E}=\frac{4\sqrt{{\left(\sum_{i=1}^{k}\sqrt{N_{i}}\sin(\theta_{i})\right)}^{2}+{\left(\sum_{i=1}^{k}\sqrt{N_{i}}\cos(\theta_{i})\right)}^{2}}}{\sum_{i=1}^{k}\sqrt{N_{i}}}$$

Using the fact that the variance of $\sqrt{N_{i}}$ is approximately $\frac{1}{4}$, he shows that the approximate variance for $\hat{\alpha }$_*E *_is 2/*N *where *N *= Σ*N*_*i*_. Edwards estimates *R *by replacing *α *with $\hat{\alpha }$_*E*_, i.e.,

$${\hat{R}}_{E}=\frac{1+{\hat{\alpha }}_{E}}{1-{\hat{\alpha }}_{E}}.$$

In the subsequent sections, we borrow this geometric framework todevelop alternative estimators of *α *and *R*.

### Moment-based Estimation of a Using Non-transformed Data

We consider two new estimators of *α*. Instead of basing these on square-root transformed data, we use the data in their original scale. The first estimator of *α *that we consider depends on the distance from the origin to the center of gravity of *k *masses of weight *N*_*i *_each placed on the rim of a unit circle in direction *θ*_*i *_= 2*πi*/*k*, i.e.,

$$D=\sqrt{{\left(\frac{1}{k}\sum_{i=1}^{k}N_{i}\sin(\theta_{i})\right)}^{2}+{\left(\frac{1}{k}\sum_{i=1}^{k}N_{i}\cos(\theta_{i})\right)}^{2}}.$$

Let *D*_*y *_= $\frac{1}{k}\sum_{i=1}^{k}N_{i}$ sin(*θ*_*i*_) be the vertical component and *D*_*x *_= $\frac{1}{k}\sum_{i=1}^{k}N_{i}$ cos(*θ*_*i*_) be the horizontal component of the distance from origin to the center of gravity of the *k *masses. Let *N *= Σ*N*_*i*_. From the exact expressions for *E*[*D*_*x*_|*N*] and *E*[*D*_*y*_|*N*] (see Additional file 1), a first-order approximation for *E*[*D*|*N*] is given by:

$$E[D|N]\approx \sqrt{E{\left[D_{x}|N\right]}^{2}+E{\left[D_{y}|N\right]}^{2}}=\frac{N\alpha }{2k}.$$

Setting *D *equal to *E*[*D*|*N*] and solving for *α *yields the following moment-based estimator for *α*:

(2) $${\hat{\alpha }}_{D}=\frac{2kD}{N}.$$

This estimator is the same as Nam's locally reasonable estimator [14]. It can also be derived from least-squares estimation of the parameters of the periodic model:

(3) *N*_*i *_= *β*_0 _+ *β*_1 _sin(*θ*_*i*_) + *β*_2 _cos(*θ*_*i*_) + *ε*_*i*_,

from which *R *is estimated as:

$${\hat{R}}_{LS}=\frac{{\hat{\beta }}_{0}+\sqrt{{\hat{\beta }}_{1}^{2}+{\hat{\beta }}_{2}^{2}}}{{\hat{\beta }}_{0}-\sqrt{{\hat{\beta }}_{1}^{2}+{\hat{\beta }}_{2}^{2}}}.$$

This relation also suggests a two-step weighted least-squares estimator of *R*. In the first step, least squares is used to estimate the parameters in (3) and then predicted values of each ${\hat{N}}_{i}$ are generated. In the second step, the parameters of (3) are estimated using weighted least squares with weights given by *w*_*i *_= 1/${\hat{N}}_{i}$. The optimality of these weights assumes that the variance of *N*_*i *_is equal to the expected value of *N*_*i*_. This procedure could be iterated until the estimates and weights converge.

The second estimator of *α *that we consider is based on the second-order moment statistic *D*^2^. This statistic is appealing because we can express the expected value of *E*[*D*^2^|*N*] exactly, whereas *E*[*D*|*N*] is only available to a first-order approximation. Using the exact expressions for $E[D_{y}^{2}|N]$ and $E[D_{x}^{2}|N]$ (see Additional file 1), we see that

$$E[D^{2}|N]=E[D_{x}^{2}|N]+E[D_{y}^{2}|N]=\frac{N}{k^{2}}\left\{1-\frac{\alpha^{2}}{4}+\frac{\alpha^{2}N}{4}\right\}.$$

Solving this expression for *α *yields the estimator

$${\hat{\alpha }}^{*}=2\sqrt{\frac{D^{2}k^{2}-N}{N(N-1)}}.$$

When *D*^2 ^is less than *N*/*k*^2 ^(the expected value of *E*[*D*^2^|*N*] at *α *= 0), this estimator results in invalid (imaginary) estimates of *α*. To remedy this, we propose the following modified estimator

$${\hat{\alpha }}_{D2}=2\sqrt{\frac{D^{2}k^{2}-Nf}{N(N-1)}},$$

where

$$f=\frac{D^{2}k^{2}/N}{1+D^{2}k^{2}/N}.$$

This modification insures that the quantity inside the square root is always greater than or equal to zero. For small values of *D*^2^, ${\hat{\alpha }}_{D2}\approx {\hat{\alpha }}_{D}$. As *D*^2 ^increases, *f *converges to 1 and $\hat{\alpha }$_*D*2 _corresponds to the estimator using the exact expression for *E*[*D*^2^|*N*].

Given an estimate of *α*, *R *can be estimated by substituting $\hat{\alpha }$ into the formula that relates *R *to *α*:

$$\hat{R}=\frac{1+\hat{\alpha }}{1-\hat{\alpha }}.$$

Ratio estimators such as $\hat{R}$ are known to be biased upwards, particularly with sparse data. Later we discuss a bias-correction term for this estimate of *R*.

### Confidence Intervals for *R*

Constructing confidence intervals for *R *is problematic because the null value lies on the boundary of the points of support for *R*. Frangakis and Varadhan recently proposed an approach for computing exact confidence limits for the seasonal relative risk derived from simulation and maximum likelihood estimation of parameters in a circular normal probability model.[19] Their approach can be adapted to estimate confidence intervals for any of the moment estimators proposed in this paper.

The approach involves finding the roots of the function *h*(*R*) = |$\hat{R}$ - *R*| - *q*(*R*; *α*), where *q*(*R*; *α*) is the 1 - *α *quantile of |$\hat{R}$ - *R*|. Note that *q*(*R*; *α*) depends on a particular estimator, although we do not make this explicit in the notation. The lower confidence limit is either zero or the value of the smaller root, whichever is larger. The upper confidence limit is the value of the larger root. Since *q *cannot be expressed in closed form, it is estimated via simulation. For a given value of *R*, data are simulated from the probability model and |$\hat{R}$ - *R*| is computed for each simulated data set. In the simulation, the parameter *φ *can be held fixed at its estimated value. The value of *q*(*R*; *α*) is then estimated by taking the empirical 1 - *α *quantile of the simulated values of *q*. The roots of *h *can be found by using an iterative algorithm.

For the estimators considered in this paper, it is possible that the function *h *will have only one root. This situation occurs when the number of events is small and/or the seasonality is strong enough so that no upper bound can be placed on the strength of seasonality (the fitted trough of the sine curve is close to zero). When only a single root is found, we set the upper confidence limit to infinity.

While this approach yields confidence intervals that are correct under the assumed probability model, it is computationally intensive and requires specialized software. We also consider a simple *ad hoc *approach for the estimation of approximate confidence limits for *R*. This approach is based on a normal approximation to the sampling distribution of log($\hat{R}$). We enforce the boundary constraint by truncating the lower confidence limit at one. This procedure yields a lower limit given by:

$${\hat{R}}_{L}=\max\left(\exp\left[\ln(\hat{R})-Z_{1-\alpha /2}\hat{\text{SE}}(\ln(\hat{R}))\right],1\right).$$

The upper limit is unbounded and given by

$${\hat{R}}_{U}=\exp\left[\ln(\hat{R})-Z_{1-\alpha /2}\hat{\text{SE}}(\ln(\hat{R}))\right].$$

A first-order Taylor series approximation for the standard error for the sampling distribution of log($\hat{R}$) is given by

$$\hat{\text{SE}}(\ln(\hat{R}))\approx \frac{2\sqrt{\text{V}\hat{\text{A}}\text{R}}(\hat{\alpha })}{\left(1+\hat{\alpha })(1-\hat{\alpha }\right)}.$$

For all estimators, $\text{V}\hat{\text{A}}\text{R}(\hat{\alpha })\approx 2/N$.

## Simulation Study

We compared the various estimators discussed in this paper in a comprehensive Monte Carlo simulation study. Initially, we set *k *= 12 (corresponding to monthly observations) with *n *= 150, *n *= 500, and *n *= 2500. For each setting of *k *and *n*, we simulated data for values of *R *ranging from 1.05 to 3.05 in increments of 0.25.

For each simulated data set, we evaluate the following five estimators of *R*:

1. $\hat{R}$_*E*_: an estimate of $\hat{R}$ using Edwards's estimator of *α*,

2. $\hat{R}$_*LS*_: an estimate of *R *using least squares,

3. $\hat{R}$_*D*2_: an estimate of *R *using $\hat{\alpha }$_*D*2_,

4. $\hat{R}$_*WLS*_: an estimate of *R *using weighted least squares,

5. $\hat{R}$_*MLE*_: an estimate of *R *using the maximum likelihood estimate of *α*.

We consider various perturbations of these baseline parameters in sensitivity analyses. First, we set *k *= 52 (corresponding to weekly observations) with *n *= 1000, *n *= 5000 and *n *= 10000. We also simulated data under two different probability models that departed from the assumed model: 1) a negative binomial model with the mean given by Edwards's model (1), but in which the counts were overdispersed with variance given by VAR[*N*_*i*_] = 1.5*E*[*N*_*i*_]; and 2) a model that generated data with a marginal mean given by Edwards's model, but in which the counts were strongly autocorrelated and overdispersed. We created autocorrelation and overdispersion among the observations by simulating *N*_1 _using Edwards's model, and then generating each *N*_*i*_, *i *= 2, ..., *k *by simulating *Q*_*i *_from Edwards model and then letting *N*_*i *_= *Q*_*i *_+ 0.1{*E*[*N*_*i*-1_] - *N*_*i*-1_}.

Additionally, we use the simulation results to evaluate the adequacy of the *ad hoc *confidence interval procedure suggested in section 2.4. For each simulated data set, we compute a 95% confidence interval for $\hat{R}$_*D*2_, $\hat{R}$_*WLS*_, and $\hat{R}$_*MLE *_and record the relative frequency of estimated confidence intervals that contain the true parameter.

### Computation

All simulations were performed in SAS V9.1 running on a Windows XP platform using software created by the authors. The maximum likelihood estimates were found using PROC NLMIXED in which the likelihood function (conditional on *N*) is maximized using a Newton-Raphson algorithm with a line search and boundary constraint (see Additional file 2 for example program). For the Monte Carlo simulation study, the true parameter value was used as the starting point for the maximization routine. The weighted least-squares estimates were obtained in a two-step procedure using PROC GENMOD.

### Results

In table 1, we report the bias and MSE from the baseline simulation. For all values of *n *and *R*, the new estimator $\hat{R}$_*D*2 _had the smallest bias of all those considered. For *n *= 150, $\hat{R}$_*D*2 _also had the smallest MSE for all values of *R*. For *n *= 500 and *n *= 2500, $\hat{R}$_*D*2 _had minimal or close to minimal MSE for smaller values of *R *(*R *< 1.85); however, for large values of *R*, $\hat{R}$_*WLS *_and $\hat{R}$_*MLE *_were better from the MSE perspective. The MSE of the estimator $\hat{R}$_*LS *_was similar, but sometimes slightly larger, than that of $\hat{R}$_*WLS*_. Edwards's estimator was the most biased and had the largest MSE for all values of *n *and *R*. All estimators evaluated were biased upwards for values of *R *close to unity, a consequence of the behavior of the estimators near the boundary.

**Table 1:** Estimated bias and MSE for each estimator from the baseline simuala-tion for *n *= 150, 500, and 2500 based on 1,000 simulated datasets

	*n *= 150									
	BIAS × 10					MSE × 10				
True R	$\hat{R}$_*D*2_	$\hat{R}$_*LS*_	$\hat{R}$_*WLS*_	$\hat{R}$_*MLE*_	$\hat{R}$_*E*_	$\hat{R}$_*D*2_	$\hat{R}$_*LS*_	$\hat{R}$_*WLS*_	$\hat{R}$_*MLE*_	$\hat{R}$_*E*_
1.05	1.93	3.07	3.07	3.12	3.17	0.85	1.51	1.51	1.55	1.63
1.30	0.63	1.87	1.86	2.05	2.04	0.90	1.32	1.30	1.38	1.51
1.55	0.23	1.59	1.57	1.65	1.94	1.51	1.90	1.84	1.83	2.32
1.80	0.16	1.63	1.60	1.62	2.28	2.35	2.82	2.66	2.63	3.86
2.05	0.18	1.75	1.68	1.72	2.84	3.31	3.94	3.65	3.71	6.29
2.30	0.37	2.06	1.99	1.99	3.85	4.68	5.62	5.04	5.01	10.06
2.55	0.60	2.44	2.33	2.34	5.18	6.30	7.67	6.72	6.85	17.16
2.80	0.83	2.84	2.66	2.69	7.35	8.53	10.51	8.94	9.22	46.54
3.05	1.10	3.30	3.03	3.06	10.27	11.34	14.20	11.67	12.29	193.5

	*n *= 500									
	BIAS × 10					MSE × 10				
True R	$\hat{R}$_*D*2_	$\hat{R}$_*LS*_	$\hat{R}$_*WLS*_	$\hat{R}$_*MLE*_	$\hat{R}$_*E*_	$\hat{R}$_*D*2_	$\hat{R}$_*LS*_	$\hat{R}$_*WLS*_	$\hat{R}$_*MLE*_	$\hat{R}$_*E*_

1.05	0.75	1.29	1.29	1.33	1.30	0.16	0.28	0.28	0.30	0.29
1.30	-0.07	0.49	0.49	0.52	0.53	0.26	0.28	0.28	0.28	0.29
1.55	-0.13	0.40	0.40	0.41	0.52	0.43	0.44	0.43	0.42	0.48
1.80	-0.10	0.41	0.41	0.41	0.66	0.62	0.63	0.61	0.61	0.73
2.05	-0.07	0.45	0.44	0.44	0.93	0.85	0.88	0.85	0.84	1.12
2.30	-0.05	0.49	0.47	0.47	1.29	1.14	1.19	1.12	1.12	1.66
2.55	-0.02	0.55	0.52	0.52	1.80	1.51	1.58	1.47	1.47	2.48
2.80	0.03	0.63	0.59	0.60	2.47	1.97	2.08	1.90	1.90	3.70
3.05	0.08	0.72	0.67	0.67	3.31	2.52	2.67	2.40	2.40	5.45

	*n *= 2500									
	BIAS × 10					MSE × 10				
True R	$\hat{R}$_*D*2_	$\hat{R}$_*LS*_	$\hat{R}$_*WLS*_	$\hat{R}$_*MLE*_	$\hat{R}$_*E*_	$\hat{R}$_*D*2_	$\hat{R}$_*LS*_	$\hat{R}$_*WLS*_	$\hat{R}$_*MLE*_	$\hat{R}$_*E*_
1.05	1.53	3.81	3.81	4.07	3.84	0.22	0.35	0.34	0.37	0.35
1.30	-0.64	0.89	0.87	0.86	1.02	0.58	0.55	0.55	0.55	0.57
1.55	-0.44	0.74	0.71	0.71	1.35	0.84	0.83	0.81	0.81	0.88
1.80	-0.32	0.76	0.70	0.69	2.40	1.19	1.20	1.14	1.15	1.35
2.05	-0.23	0.84	0.75	0.75	4.23	1.65	1.66	1.56	1.56	2.06
2.30	-0.13	0.97	0.85	0.82	6.98	2.22	2.24	2.07	2.07	3.18
2.55	0.00	1.15	0.97	0.92	10.77	2.93	2.96	2.69	2.70	4.94
2.80	0.16	1.37	1.15	1.11	15.76	3.79	3.83	3.43	3.44	7.73
3.05	0.28	1.58	1.28	1.24	21.98	4.80	4.86	4.27	4.29	11.99

In the sensitivity analyses in which we generated overdispersed and auto-correlated data, the same essential patterns prevailed. The bias of $\hat{R}$_*D*2 _was minimal for all values of *R *in each scenario. Edwards's estimator was the most biased and had the largest MSE for all values of *n *and *R*. In these simulations that depart from the assumed model, the MSE of $\hat{R}$_*WLS *_was better than $\hat{R}$_*MLE *_for certain values of *R *and *n*. This result is likely due to the fact that the MLE is not based on the probability model used to generate the data. In table 2, we report the MSE of $\hat{R}$_*D*2 _relative to $\hat{R}$_*WLS *_for the overdispersed and auto correlated data-generating distributions, respectively. In these figures, relative MSEs below 1 indicate that $\hat{R}$_*D*2 _is preferable from the MSE perspective. Both figures reveal that the relative MSE increases with *R*. For small values of *R *and *n*, $\hat{R}$_*D*2 _is preferable. For larger values of *R*, $\hat{R}$_*WLS *_was preferable. These results were the most pronounced in the setting of autocorrelated data. The MSE of $\hat{R}$_*D*2 _was never more than 13% greater than $\hat{R}$_*WLS*_; however, it was nearly half as much for small values of *R*. For the simulations in which *k *= 52, the estimator $\hat{R}$_*D*2 _continued to be the least biased, but there was little difference in MSE between $\hat{R}$_*D*2_, $\hat{R}$_*WLS*_, and $\hat{R}$_*MLE *_in terms of MSE across all values of *R*.

**Table 2:** Relative mean squared error of $\hat{R}$_*D*2 _to $\hat{R}$_*WLS*_

	Overdispersed			Autocorrelated		
True R	*n *= 150	*n *= 500	*n *= 2500	*n *= 150	*n *= 500	*n *= 2500
1.05	0.64	0.66	0.69	0.69	0.61	0.66
1.30	0.71	0.88	1.03	1.03	0.93	1.05
1.55	0.82	0.96	1.01	1.01	1.00	1.04
1.80	0.91	0.96	1.00	1.00	1.01	1.04
2.05	0.92	0.97	1.01	1.01	1.01	1.06
2.30	0.94	0.97	1.02	1.02	1.02	1.07
2.55	0.99	0.98	1.02	1.02	1.03	1.09
2.80	1.01	0.99	1.03	1.03	1.04	1.11
3.05	1.07	1.00	1.04	1.04	1.07	1.13

In table 3, we report the estimated coverage probabilities for the *ad hoc *confidence intervals computed for the estimators $\hat{R}$_*D*2_, $\hat{R}$_*LS*_, and $\hat{R}$_*WLS*_. The actual coverage probabilities are close to correct, usually within one to two percentage points of the nominal 95%. The coverage probabilities for these confidence intervals in the setting of autocorrelation and overdispersion was substantially lower, with actual coverage probabilities ranging from 87% to 96%.

**Table 3:** Percentage of estimated *ad hoc *95% confidence intervals that cover the true parameter

	*n *= 150			*n *= 500			*n *= 2500		
True R	$\hat{R}$_*D*2_	$\hat{R}$_*LS*_	$\hat{R}$_*MLE*_	$\hat{R}$_*D*2_	$\hat{R}$_*LS*_	$\hat{R}$_*MLE*_	$\hat{R}$_*D*2_	$\hat{R}$_*LS*_	$\hat{R}$_*MLE*_
1.05	95.1	91.6	92.6	96.1	92.4	92.9	97.8	95.7	95.4
1.30	98.3	96.2	97.0	97.8	97.2	97.4	93.6	96.0	95.9
1.55	98.9	97.5	98.2	95.1	97.3	97.7	94.2	95.4	95.5
1.80	97.1	97.1	98.4	95.7	96.1	96.9	94.3	94.8	95.2
2.05	96.3	97.5	98.4	95.8	95.7	96.8	94.8	94.8	95.1
2.30	95.8	96.9	98.2	95.9	95.9	96.9	94.8	94.8	95.2
2.55	95.8	96.7	98.1	95.7	95.9	97.1	95.3	94.4	95.5
2.80	95.8	96.2	98.0	96.4	96.1	96.9	95.5	94.5	95.9
3.05	96.2	96.8	98.1	96.5	96.1	97.1	95.4	94.7	96.3

As a side note, the algorithm that we used to find the MLE experienced convergence problems close to *R *= 1. For *R *= 1.05, the MLE failed to converge in roughly 20% of the simulated data sets. This problem diminished as *R *increased. For *R *= 1.5 the MLE was located for 95% of the simulated data sets. This is likely to be a result of near non-identifiability of *φ *when the seasonality is weak. More computationally-intensive approaches, such as a grid search, might alleviate this problem; however, in the context of a simulation study, we required an approach that could converge rapidly. For all results discussed below, we excluded simulated data sets for which the MLE was not found. We found that the simulation results for the non-missing estimators were largely unaffected by the inclusion/exclusion of the simulations for which the MLE was not located.

### Example: Seasonality of Monocytic Leukemia

We compared the estimators proposed in this paper with the MLE and the estimator of Edwards through a re-analysis of data on the seasonal incidence of monocytic leukemia in England and Wales from 1974–1998 (*N *= 2311, *k *= 12) with monthly counts given as (203, 203, 197, 206, 204, 216, 165, 161, 177, 179, 200, 200). We used data from the Office of National Statistics as reported by Eatough [7]. In Table 4, we report the point estimate and approximate 95% confidence limits corresponding to each of the five estimators considered in the simulation study. We also present the confidence limits computed using the method of Frangakis and Varadhan [19]. These confidence intervals could not be computed for the MLE because the convergence problems experienced by the maximization algorithm made the computation of *q *infeasible.

**Table 4:** Estimated peak-to-low ratio and 95% CI for the seasonal incidence of monocytic leukemia in England and Wales (1974–98) using four different estimators and two confidence interval procedures

		*Ad hoc* 95% CL		Method of F & V 95% CL	
Estimator	Point Estimate	Lower Limit	Upper Limit	Lower Limit	Upper Limit
$\hat{R}$_*E*_	1.20	1.07	1.35	1.07	1.37
$\hat{R}$_*LS*_	1.20	1.06	1.34	1.07	1.36
$\hat{R}$_*D*2_	1.18	1.05	1.32	1.07	1.33
$\hat{R}$_*MLE*_	1.20	1.07	1.35	*	*

The different estimators do not lead to substantively different interpretations of the data. Nevertheless, consistent with the results of the simulation, the estimators $\hat{R}$_*D*2 _are smaller than *R*_*LS *_and Edwards estimator. Given the large number of events and the fact that the data exhibit only moderate seasonality, the simulation study suggests that Edwards estimator should be only moderately biased for these data. The confidence intervals computed by the *ad hoc *confidence interval procedure were nearly identical to those of Frangakis and Varadhan.

## Discussion

In this paper we have proposed a new estimator of the peak-to-low ratio of a periodic process and compared it to several alternative estimators, including Edwards's estimator, the MLE, and weighted least squares. Studies employing Edwards's method often involve very rare events and moderate seasonality. For these studies, the estimator proposed in this paper appears to be optimal. It has less bias and a smaller MSE than any of the estimators considered, including the MLE and weighted least squares. Weighted least squares was preferable from a MSE perspective in the setting of frequent outcomes or strong seasonality. We speculate that the simple estimator proposed in this paper improves upon the estimator of Edwards and the other moment-based estimator because it is based on an exact rather than an approximate expression for the distance from the origin to the center of gravity. We further speculate that the bias and inefficiency in the MLE is due to the small event rates considered in this paper.

The *ad hoc *confidence interval procedure that we evaluated performed reasonably well for data generated from Edwards's probability model. If more precise confidence intervals are needed, the computationally-intensive approach proposed by Frangakis and Varadhan can be employed [19]. Users should be aware that both of the confidence intervals considered in this paper are model based. If the underlying model is wrong, for example, in the setting of strongly autocorrelated or overdispersed data, the true coverage probabilities may differ from the nominal 95%.

Because ratio estimators are known to be biased upwards, particularly with sparse data, we also considered a bias-corrected estimator based on the expected value of a second-order Taylor series expansion of (1 + $\hat{\alpha }$)/(1 - $\hat{\alpha }$) around *α *given by

$$\begin{array}{c} E\left[\frac{1+\hat{\alpha }}{1-\hat{\alpha }}\right]\approx \frac{1+\alpha }{1-\alpha }+\frac{2\text{VAR}[\hat{\alpha }]}{{\left(1-\alpha \right)}^{3}}\\ =\frac{1+\alpha }{1-\alpha }\left(1+\frac{2\text{VAR}[\hat{\alpha }]}{(1+\alpha ){\left(1-\alpha \right)}^{2}}\right). \end{array}$$

This approximation led to the following bias-corrected estimator of *R*:

(4) $$\hat{R}=\frac{1+\hat{\alpha }}{1-\hat{\alpha }}{\left(1+\frac{2\text{V}\hat{\text{A}}\text{R}[\hat{\alpha }]}{(1+\hat{\alpha }){\left(1-\hat{\alpha }\right)}^{2}}\right)}^{-1}.$$

We found that estimators based on this correction factor tended to be somewhat over-corrected, possibly because they are based on an approximation of the variance of $\hat{\alpha }$.

One important limitation of the estimators proposed in this paper is that they are based on the assumption of a single cyclical effect (harmonic) that can be well approximated by a sine curve. For more complex data, with multiple periodic components or a linear trend, alternative statistical methods should be used. For such data there exist more complex harmonic models [20,12], spectral methods [21], and various periodic regression models. Also, we outline an approach to estimating seasonal intensity using a periodic generalized linear model that assumes a log link and a Poisson distributed outcome (see Additional file 3). This approach is based on a different model for the mean, i.e., that the log of the expected value of the counts is a sinusoidal function. However, it allows for the inclusion of covariates and extends naturally to variably-sized intervals through use of a Poisson offset.

Edwards's method has been widely used in epidemiology in studies of seasonality. In this paper we have shown that Edwards's estimator of the seasonal relative risk can be substantially biased. The estimator proposed in this paper represents a straightforward modification of Edwards's estimator. Like that of Edwards, it is a simple estimator that is available in closed form. For modest seasonality and small numbers of events, this estimator appears to have the best finite sample performance characteristics of those estimators considered.

For more frequent events or stronger seasonality, the weighted least-squares approach discussed in this paper is preferable and is easily implemented using standard statistical software.

## Competing interests

The authors declare that they have no competing interests.

## Authors' contributions

KR conceived the project. Both authors contributed equally to evaluation and development of statistical methodology. MB carried out programming, simulation, and data analysis. MB drafted the manuscript. Both authors read and approved the final manuscript.

## Pre-publication history

The pre-publication history for this paper can be accessed here:



## Supplementary Material

Additional file 1 **Derivations.** The file provides mathematical derivations of several expressions referenced in the paper. Click here for file

Additional file 2 **SAS Program.** This file provides the SAS program used to located themaximum likelihood estimate of Edwards's model. Click here for file

Additional file 3 **Periodic generalized linear model approach to estimating seasonal intensity**. The file outlines an approach to estimating the peak-to-low ratio using a periodic generalized linear model. Click here for file
