# Supplementary material for: Simple estimators of the intensity of seasonal occurrence
Source: BMC Med Res Methodol. 2008 Oct 22;8:67. doi: 10.1186/1471-2288-8-67 (PMC2596789; doi:10.1186/1471-2288-8-67)
Supplement: Additional file 1 — Derivations. The file provides mathematical derivations of several expressions referenced in the paper. [file 1471-2288-8-67-S1.pdf]

Additional File 1: Derivations

For notational simplicity let $\theta_i = 2\pi(i-0.5)/k$ and $\gamma = -2\pi\phi/k$. Our results use the identities,

$$\sum_{i=1}^{k} \sin(\theta_i) = \sum_{i=1}^{k} \cos(\theta_i) = \sum_{i=1}^{k} \sin(\theta_i)\cos(\theta_i) = \sum_{i=1}^{k} \sin^2(\theta_i)\cos(\theta_i)$$

$$= \sum_{i=1}^{k} \sin(\theta_i)\cos^2(\theta_i) = \sum_{i=1}^{k} \sin^3(\theta_i) = \sum_{i=1}^{k} \cos^3(\theta_i) = 0,$$

and

$$\sum_{i=1}^{k} \sin^2(\theta_i) = \sum_{i=1}^{k} \cos^2(\theta_i) = \frac{k}{2}.$$

Conditional on N, the counts N_i are multinomial with probabilities

$$P_i = \frac{1}{k}(1 + \alpha\cos(\theta_i + \gamma)),$$

$i = 1, \ldots, k$ and size N.

The expected value of the vertical component of the distance from the origin to the center of gravity conditional on the total number of observed events N is given by

$$E[D_y|N] = \frac{1}{k}\sum_{i=1}^{k} NP_i\sin(\theta_i) = \frac{N}{k^2}\sum_{i=1}^{k}(1 + \alpha\cos(\theta_i + \gamma))\sin(\theta_i)$$

$$= \frac{N}{k^2}\sum_{i=1}^{k}(1 + \alpha\cos(\theta_i)\cos(\gamma) - \alpha\sin(\theta_i)\sin(\gamma))\sin(\theta_i)$$

$$= -\frac{N}{k^2}\sum_{i=1}^{k}\alpha\sin^2(\theta_i)\sin(\gamma) = -\frac{N\alpha}{2k}\sin(\gamma).$$

Similarly, the conditional expectation of the horizontal component of the distance from the origin to the center of gravity is given by:

$$E[D_x|N] = \frac{N\alpha}{2k}\cos(\gamma).$$

The conditional expected value of the squared vertical component of the distance from the origin to the center of gravity is given by

$$E[D_y^2|N] = E[\left\{\frac{1}{k}\sum_{i=1}^{k} N_i\sin(\theta_i)\right\}^2|N] = \frac{1}{k^2}\sum_{i=1}^{k}\sum_{j=1}^{k} E[N_iN_j|N]\sin(\theta_i)\sin(\theta_j)$$

$$= \frac{1}{k^2}\left\{\sum_{i=1}^{k}\sum_{j=1}^{k} COV[N_i, N_j|N]\sin(\theta_i)\sin(\theta_j) + \sum_{i=1}^{k}\sum_{j=1}^{k} E[N_i|N]E[N_j|N]\sin(\theta_i)\sin(\theta_j)\right\}$$

$$= \frac{1}{k^2}\left\{\sum_{i=1}^{k} NP_i\sin^2(\theta_i) - \sum_{i=1}^{k}\sum_{j=1}^{k} NP_iP_j\sin(\theta_i)\sin(\theta_j) + \sum_{i=1}^{k}\sum_{j=1}^{k} N^2P_iP_j\sin(\theta_i)\sin(\theta_j)\right\}.$$

where

$$\sum_{i=1}^{k} NP_i\sin^2(\theta_i) = \frac{N}{k}\sum_{i=1}^{k}(1+\alpha\cos(\theta_i+\gamma))\sin^2(\theta_i)$$

$$= \frac{N}{k}\sum_{i=1}^{k}(1+\alpha\cos(\theta_i)\cos(\gamma) - \alpha\sin(\theta_i)\sin(\gamma))\sin^2(\theta_i) = \frac{N}{k}\sum_{i=1}^{k}\sin^2(\theta_i) = \frac{N}{2}$$

and

$$\sum_{i=1}^{k}\sum_{j=1}^{k} NP_iP_j\sin(\theta_i)\sin(\theta_j) = N\sum_{i=1}^{k} P_i\sin(\theta_i)\left\{\sum_{j=1}^{k} P_j\sin(\theta_j)\right\},$$

$$= \frac{k^2E[D_y|N]^2}{N} = \frac{N\alpha^2}{4}\sin^2(\gamma),$$

and

$$\sum_{i=1}^{k}\sum_{j=1}^{k} E[N_i|N]E[N_j|N]\sin(\theta_i)\sin(\theta_j) = \sum_{i=1}^{k} E[N_i|N]\sin(\theta_i)\left\{\sum_{j=1}^{k} E[N_j|N]\sin(\theta_j)\right\},$$

$$= k^2E[D_y|N]^2 = \left(-\frac{N\alpha}{2}\sin(\gamma)\right)^2;$$

so

$$E[D_y^2|N] = \frac{1}{k^2}\left\{\frac{N}{2} - \frac{N\alpha^2}{4}\sin^2(\gamma) + \frac{N^2\alpha^2}{4}\sin^2(\gamma)\right\}.$$

Likewise, the same algebraic steps show the expected value of the squared horizontal component of the distance from the origin to the center of gravity is given by:

$$E[D_x^2|N] = \frac{1}{k^2}\left\{\frac{N}{2} - \frac{N\alpha^2}{4}\cos^2(\gamma) + \frac{N^2\alpha^2}{4}\cos^2(\gamma)\right\}.$$
