# Supplementary material for: Simple estimators of the intensity of seasonal occurrence
Source: BMC Med Res Methodol. 2008 Oct 22;8:67. doi: 10.1186/1471-2288-8-67 (PMC2596789; doi:10.1186/1471-2288-8-67)
Supplement: Additional file 2 — SAS Program. This file provides the SAS program used to located themaximum likelihood estimate of Edwards's model. [file 1471-2288-8-67-S2.pdf]

Additional File 2: SAS Code

The maximum likelihood estimates are found using PROC NLIMIXED in SAS.

```
proc nlmixed tech=newrap lis=3 lsp=0.0001;
parms phi=0 logR=0;
bounds 0<=logR;
R=exp(logR);
alpha=(R-1)/(R+1);
mu=(1/12)*(1+alpha*cos((6.283185308/12)*(t-phi-0.5)));
    ll=log(mu)*y;
model y~general(ll);
run;
```
