# Supplementary material for: Simple estimators of the intensity of seasonal occurrence
Source: BMC Med Res Methodol. 2008 Oct 22;8:67. doi: 10.1186/1471-2288-8-67 (PMC2596789; doi:10.1186/1471-2288-8-67)
Supplement: Additional file 3 — Periodic generalized linear model approach to estimating seasonal intensity. The file outlines an approach to estimating the peak-to-low ratio using a periodic generalized linear model. [file 1471-2288-8-67-S3.pdf]

Additional File 3: Periodic Generalized Linear Model Approach to Estimating Seasonal Intensity

One motivation for this work was to explore alternative estimators of seasonal intensity that might improve on Edwards's estimator but would not depend on iterative algorithms and thus could be included in an Excel spreadsheet-based collection of epidemiological analysis tools [15]. However, estimates of seasonal intensity can also be obtained using a generalized linear model (GLM) framework that is estimable in all modern statistical software packages. This approach requires one to specify a different model for the mean, but leads to straightforward estimation in the GLM framework.

The natural application of the GLM approach to the estimation of seasonal intensity is to assume that the counts are Poisson distributed with mean

$$\log(E[N_i]) = \beta_0 + \beta_1 \sin(\theta_i) + \beta_2 \cos(\theta_i).$$

The peak-to-low ratio of the process is given by

$$R = \frac{\exp\{\beta_0 + \sqrt{\beta_1^2 + \beta_2^2}\}}{\exp\{\beta_0 - \sqrt{\beta_1^2 + \beta_2^2}\}} = \exp\{2\sqrt{\beta_1^2 + \beta_2^2}\},$$

and thus R can be estimated with:

$$\hat{R}_{GLM} = \exp\{2\sqrt{\hat{\beta}_1^2 + \hat{\beta}_2^2}\}.$$

The approximate variance for R_{GLM} can be computed using the Delta method. Confidence intervals could be computed using the either of the approaches discussed in this paper.

The GLM approach allows for the inclusion of covariates and additional periodic components of different frequencies. Also, by setting the value of the Poisson offset, one can allow the intervals to be of different lengths or one can allow the size of the population at risk to vary. However, the expected counts under the GLM model are no longer a pure sinusoidal function, but rather the log of the expected values are assumed to be sinusoidal. For weak to moderate seasonality, the two models are similar. For strong seasonality, the two models have a substantially different shape and subject matter expertise or model fit diagnostics should be used to determine which model is more appropriate.
